# Levels of Cocaine- and Amphetamine-Regulated Transcript in Vagal Afferents in the Mouse Are Unaltered in Response to Metabolic Challenges

**DOI:** 10.1523/ENEURO.0174-16.2016

**Published:** 2016-10-05

**Authors:** Xuefeng Yuan, Ying Huang, Sarita Shah, Hua Wu, Laurent Gautron

**Affiliations:** 1 Division of Hypothalamic Research and Department of Internal Medicine, The University of Texas Southwestern Medical Center, 5323 Harry Hines Blvd., Dallas, Texas 75390; 2 Department of Orthopedics, Tongji Hospital, Tongji Medical College, Huazhong University of Science and Technology, Wuhan, China

**Keywords:** immunohistochemistry, metabolism, neuropeptide, vagus nerve

## Abstract

Cocaine- and amphetamine-regulated transcript (CART) is one of the most abundant neuropeptides in vagal afferents, including those involved in regulating feeding. Recent observations indicate that metabolic challenges dramatically alter the neuropeptidergic profile of CART-producing vagal afferents. Here, using confocal microscopy, we reassessed the distribution and regulation of CART(55–102) immunoreactivity in vagal afferents of the male mouse in response to metabolic challenges, including fasting and high-fat-diet feeding. Importantly, the perikarya and axons of vagal C-fibers were labeled using mice expressing channelrodhopsin-2 (ChR2-YFP) in Na_v_1.8-Cre–expressing neurons. In these mice, approximately 82% of the nodose ganglion neurons were labeled with ChR2-YFP. Furthermore, ChR2-YFP–labeled axons could easily be identified in the dorsovagal complex. CART(55–102) immunoreactivity was observed in 55% of the ChR2-YFP–labeled neurons in the nodose ganglion and 22% of the ChR2-YFP–labeled varicosities within the area postrema of fed, fasted, and obese mice. The distribution of positive profiles was also identical across the full range of CART staining in fed, fasted, and obese mice. In contrast to previous studies, fasting did not induce melanin-concentrating hormone (MCH) immunoreactivity in vagal afferents. Moreover, prepro-MCH mRNA was undetectable in the nodose ganglion of fasted mice. In summary, this study showed that the perikarya and central terminals of vagal afferents are invariably enriched in CART and devoid of MCH.

## Visual Overview

**Figure:**
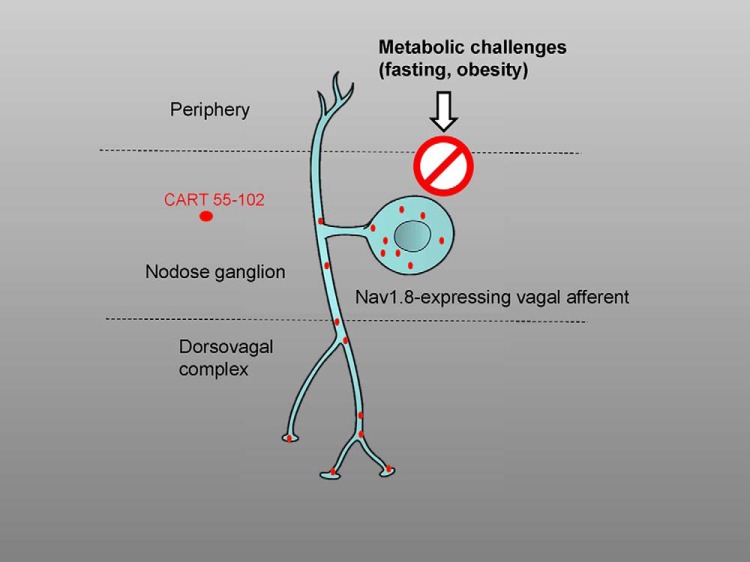

## Significance Statement

Recent studies reported that fasting triggers vagal afferents to switch from expressing anorectic to orexigenic neuropeptides. This study failed to replicate those observations using a combination of confocal microscopy, immunohistochemistry, and in situ hybridization. In particular, we showed that neither fasting nor diet-induced obesity influences immunoreactivity for cocaine- and amphetamine-regulated transcript neuropeptide in mouse vagal afferents. In contrast to previous studies, we also failed to detect melanin-concentrating hormone expression in mouse vagal afferents. Overall, we reached the conclusion that the neuropeptidergic profile of the vagal afferents involved in feeding is remarkably stable in response to metabolic challenges.

## Introduction

More than 16 years ago, Broberger et al. (1999) showed that cocaine- and amphetamine-regulated transcript (CART) was expressed in half of vagal afferents, making it one of the most abundantly expressed neuropeptides in the nodose ganglion. Different CART peptides can be generated by splicing and enzymatic cleavage of a precursor peptide in a tissue-specific manner (Koylu et al., 1997; Thim et al., 1999). The long form of CART is highly expressed in the hypothalamus and is considered to be one of the most biologically active CART peptides, with proven anorectic actions (Stanley et al., 2001). In the past, CART expression in the nodose ganglion was detected mainly using antibodies raised against the long form of CART (Broberger et al., 1999; Zheng et al., 2002).

Recent pharmacological and RNA interference experiments suggested that vagally released CART was implicated in the anorectic actions of cholecystokinin (CCK) (De Lartigue et al., 2010; Heldsinger et al., 2012). Most CART-containing vagal afferents were shown to coexpress CCK receptor (Broberger et al., 1999), further ascertaining the role of vagally released CART in feeding. In addition, one retrograde tracing study showed that many CART-positive vagal afferents project to the rat duodenum and stomach (Zheng et al., 2002). Inconsistent observations have also been reported regarding the anorectic actions of CART in the brain (Rogge et al., 2008). In particular, administration of CART in the fourth ventricle suppressed feeding (Aja et al., 2002), and CART microinjection into areas of the nucleus of the solitary tract supplied by CART-positive afferents was ineffective at suppressing feeding (Zheng et al., 2002). Hence, the role of vagally released CART in feeding is controversial. In addition, CART regulation in vagal afferents in response to metabolic challenges remains confusing. One initial study reported that the percentage of rat nodose ganglion cells expressing CART mRNA remained unchanged after food restriction or diet-induced obesity (Broberger et al., 1999). Arguing against these results, several studies reported profound alterations in the neuropeptidergic profile of CART-positive afferents upon fasting. Specifically, fasting and leptin receptor deficiency in vagal neurons were shown to suppress CART immunoreactivity in rat and mouse nodose ganglia (de Lartigue et al., 2007, 2010,2014; de La Serre et al., 2015). Furthermore, the orexigenic peptide melanin-concentrating hormone (MCH) was shown to be induced in the vagal afferents of fasted rats and mice that expressed CART in the fed condition (Burdyga et al., 2006; de Lartigue et al., 2007, 2014; de La Serre et al., 2015). To our knowledge, such a unique feeding-dependent switch in the neuropeptidergic profile of vagal afferents had never been reported before.

Given the aforementioned discrepancies and gaps in the literature, the first goal of this study was to reexamine CART(55–102) immunoreactivity in the cell body and axons of vagal C-fibers in mice submitted to different metabolic challenges. We used transgenic mice in which Na_v_1.8-expressing neurons are labeled with channelrhodopsin-2 (ChR2) fused with yellow fluorescent protein (YFP) (Na_v_1.8-Cre-ChR2-YFP mice) to identify vagal C-fibers. The second goal of this study was to characterize Na_v_1.8-Cre-ChR2-YFP mice as a useful model to label vagal C-fibers. Na_v_1.8-expressing peripheral afferents include all of the C-type peripheral afferents, as well as those expressing neuropeptides (Chiu et al., 2014; Usoskin et al., 2015). ChR2-YFP was chosen as a fluorescent reporter because of the emerging evidence of its usefulness in neuronal tract tracing studies (Jennings and Stuber, 2014). Unexpectedly, the present study showed that CART(55–102) present in the perikarya and central terminal of the vagal C-fibers was not regulated by metabolic challenges. In contrast to previous studies, we showed that CART-positive afferents in the mouse never produced MCH.

## Materials and Methods

### Generation of animals and experimental groups

Na_v_1.8-Cre-ChR2-YFP mice were generated by crossing Na_v_1.8-Cre mice [Scn10atm2(cre)Jnw], which were kindly provided to us by Dr. Wood (Stirling et al., 2005), with Ai32 mice [Ai32(RCL-ChR2(H134R)/EYFP] expressing ChR2-YFP in a Cre-dependent manner. The Ai32 mice were originally designed by the Allen Institute (Madisen et al., 2010) and are currently available from the Jackson Laboratory (stock no. 012569; Bar Harbor, ME). The mice were all adult males (ages varying between 6 and 16 weeks of age) carrying one copy of Na_v_1.8-Cre and one copy of ChR2-YFP. Na_v_1.8-Cre mice are useful for selectively inducing the expression of fluorescent proteins in C-fiber neurons, including those in the vagus nerve. The usefulness of Ai32 mice in optogenetic and tracing experiments is also well documented (Daou et al., 2013; Muñoz et al., 2014).

The experimental animals were housed in a barrier facility in a temperature-controlled environment (lights on from 0600 to 1800) with *ad libitum* access to water and standard chow with 12% of calories from fat. All animal procedures were performed in accordance with the guidelines of our Institutional Animal Care and Use Committee at the University of Texas Southwestern Medical Center. For the metabolic challenges studies, we used 22 Na_v_1.8-Cre-ChR2-YFP mice, which were divided into three groups consisting of animals fed chow *ad libitum* at all times (24.7 ± 0.9 g body weight, *n* = 7), animals fasted 16–24 h before sacrifice (21.8 ± 1.2 g body weight, *n* = 7), and animals fed a high-fat diet (62% calories from fat; Research Diet D12492i) for 4 weeks before sacrifice (35.0 ± 2.4 g body weight, *n* = 8). A separate group of Na_v_1.8-Cre-ChR2-YFP mice that were fed on chow *ad libitum* was used for the initial characterization of ChR2-YFP expression in the nodose ganglia and brains (*n* = 4), and a group of Na_v_1.8-Cre-ChR2-YFP mice was used to map the peripheral organs (*n* = 4).

Eight C56/Bl6 male mice (6 weeks of age) divided into fed and fasted groups were also used for performing *in situ* hybridization (ISH). These mice were purchased from our institution animal husbandry and housed in the same barrier facility and environment as the Na_v_1.8-Cre-ChR2-YFP mice. Immunostaining was also performed in the nodose ganglia of six lean Zucker male rats (6 months of age) that were given to us by another laboratory on our campus. Rats were housed in a conventional facility in a temperature-controlled environment with *ad libitum* access to water and standard chow. Three rats were fed chow *ad libitum* at all times, and three were fasted 16 h before sacrifice.

### Tissue collection and preparation for immunohistochemistry

On the day of sacrifice, between 0900 and 1100, the animals received an overdose of chloral hydrate (500 mg/kg, i.p.) and were then perfused transcardially with 0.9% saline followed by 10% formalin (Sigma, St. Louis, MO) for 2 min. Tissues, including the left nodose ganglion, were rapidly removed with the help of a dissecting scope and postfixed for approximately 1 h. The nodose ganglion, brain, stomach wall, and duodenum were submerged in 20% sucrose overnight at 4°C. The stomach muscularis was prepared as whole mounts for immediate immunostaining. Cryoprotected brains were sectioned at 25 μm using a freezing microtome (1:5 series). The brain sections were collected in 0.1 m PBS (pH 7.4), transferred in a cryoprotectant solution (30% sucrose, 30% ethylene glycol), and stored at −20°C. Other cryoprotected tissues were frozen in TissueTek optimum cutting temperature compound (Sakura) on dry ice before being sectioned at 16 μm using a cryostat (1:5 series). The sections were collected on SuperFrost slides and stored at −80°C.

### Antibody characterization

**Table 1** compiles the primary and secondary antibodies used in this study. A discussion of the specificity of our primary antibodies is listed below. All of them were commercially available.

**Table 1.** Information relative to the primary and secondary antisera used in the study.

**Antibody**	**Manufacturer**	**Catalog no.**	**Lot no.**	**Host**	**Working dilution**	**Immunogen**
Primary antibody						
Peripherin	EMD Millipore	AB1530	2446692	Rabbit	1:500	trp-E-peripherin fusion protein containing all but the four N-terminal amino acids of rat peripherin
CART(55–102)	Phoenix Pharmaceuticals	H-003-62	01251-10; 01251-6	Rabbit	1:800–1:1000	Ile-Pro-Ile-Tyr-Glu-Lys-Lys-Tyr -Gly-Gln-Val-Pro-Met-Cys-Asp-Ala-Gly-Glu-Gln-Cys-Ala-Val-Arg-Lys-Gly-Ala-Arg-Ile-Gly-Lys-Leu-Cys-Asp-Cys-Pro-Arg-Gly-Thr-Ser-Cys-Asn-Ser-Phe-Leu-Leu-Lys-Cys-Leu [disulfide bonds between Cys1-Cys3,Cys2-Cys5,Cys4-Cys6]
CART 1–39	Phoenix Pharmaceuticals	H-003-63	01102	Rabbit	1:800–1:1000	pGlu-Glu-Asp-Ala-Glu-Leu-Gln-Pro-Arg-Ala-Leu-Asp-Ile-Tyr-Ser-Ala-Val-Asp-Asp-Ala-Ser-His-Glu-Lys-Glu-Leu-Pro-Arg-Arg-Gln-Leu-Arg-Ala-Pro-Gly-Ala-Val-Leu-Gln
MCH	Phoenix Pharmaceuticals	H-070-47	01629-3	Rabbit	1:1000	Asp-Phe-Asp-Met-Leu-Arg-Cys-Met-Leu-Gly-Arg-Val-Tyr-Arg-Pro-Cys-Trp- Gln-Val
TH	Abcam	ab101853	GR120879-17	Goat	1:1000	Synthetic peptide corresponding to human TH(30–100) (N terminal)
ProMCH	Santa Cruz Biotechnology	sc-14509	B2415	Goat	1:100–1:1000	20-amino-acid peptide near the C-terminus of pro-MCH precursor of human origin
GFP	Aves Laboratory	GFP-10120	GFP697986	Chicken	1:1000	GFP emulsified in Freund’s adjuvant
Secondary antibody						
Anti-rabbit Alexa Fluor 594	Life Technologies	A21207	1256153	Donkey		
Anti-goat Alexa Fluor 594	Life Technologies	A11058	1608643	Donkey		
Anti-chicken Alexa Fluor 488	Life Technologies	A11039	1356650	Goat		

#### Polyclonal rabbit antibody against CART(55–102) (RRID:AB_2313614)

We used the residue nomenclature established for rat CART peptides (Rogge et al., 2008). This antiserum was extensively characterized in the central nervous system and peripheral ganglia, including the nodose ganglion (Zheng et al., 2002; Valera et al., 2006; Gonsalvez et al., 2010; Reeber and Sillitoe, 2011). Controls for specificity included preadsorption with a CART(55–102) peptide and omission of the primary antibody (Dun et al., 2000; Reeber and Sillitoe, 2011). According to the manufacturer, this antibody detects a 5-kDa band on Western blot of rat brain samples. CART staining in both the brain and nodose ganglion samples displayed a distribution pattern that perfectly agreed with previous reports using this antiserum. The neurons in the nodose ganglion were not stained when the primary antibody was omitted.

#### Antibody against CART(1–39)

We also tested an antibody against CART(1–39) (cat. no. H-003-63; Phoenix Pharmaceuticals, Burlingame, CA; cat. no. AF163; R&D Systems, Minneapolis, MN). However, this antibody failed to label the nodose ganglion and thus we have not included these data in the article. This is not completely surprising, since H-003-063 was raised against a CART fragment that is not very well studied and is not considered as biologically relevant as CART(55–102).

#### Chicken anti-GFP polyclonal antiserum

Many laboratories have used this antibody to label the mouse brain and ganglia of transgenic mice that express GFP and verified the absence of staining when it was used against wild-type tissue (Volkmann et al., 2010; Marques-Lopes et al., 2014). In addition, GFP-expressing hippocampal neurons showed endogenous fluorescence that was enhanced only by immunostaining with the antiserum (Yi et al., 2015).

#### Peripherin polyclonal antiserum

According to the manufacturer, this antiserum is regularly tested on PC12 lysates and detects a major band at 57 kDa. Lysates from cell lines transfected with the peripherin gene also showed a single band at approximately 58 kDa (Tseng et al., 2008; Xiao et al., 2008). Moreover, it was shown that the staining obtained with this antibody overlapped perfectly with that of eGFP-peripherin in PC12 cells (Tseng et al., 2008). As anticipated, this antibody produced filamentous staining of the neuronal perikarya in the nodose ganglion. Here, we used peripherin as a panneuronal marker for vagal afferents.

#### Polyclonal goat antiserum against tyrosine hydroxylase (RRID:AB_10710873)

The data from the manufacturer show that this antiserum detects a single 50-kDa band from rat, mouse, and human brain lysates. This antibody has been used to label the mouse striatum and the rat ventral tegmental area (García-Pérez et al., 2013; Biezonski et al., 2015). Omission of the antibody resulted in no staining. Here, we used the tyrosine hydroxylase (TH) antibody to label subsets of postsynaptic neurons in the dorsovagal complex. In our samples, this antiserum produced a staining pattern that was consistent with the distribution of TH-positive neurons in the mouse dorsovagal complex (Cork et al., 2015).

#### Polyclonal goat anti–pro-MCH (RRID:AB_2237276)

By Western blot, this antibody detects one band of 45–50 Kda, according to the manufacturer. Although the exact sequence of the immunogenic peptide is not available, Deurveilher et al. (2006) have discussed the observation that this antibody certainly detected MCH neurons in the lateral hypothalamus. For instance, they showed overlapping staining with another antibody and a lack of staining when the antibody was preadsorbed with the immunogen peptide (Deurveilher et al., 2006). Moreover, this antiserum did not label orexin-positive cells. In our laboratory, this antiserum exclusively labeled neurons in the lateral hypothalamus region, strongly suggesting specificity. The lateral hypothalamus was not stained when the primary antibody was omitted. Finally, another group showed that this antiserum did not immunolabel structures in mice lacking MCH neurons (Whiddon and Palmiter, 2013), further exhibiting its specificity.

#### Polyclonal rabbit antiserum against MCH (RRID:AB_10013632)

According to Deurveilher et al. (2006), this antibody labels exactly the same cells as the pro-MCH antibody described above. The manufacturer also performed competitive radioimmunoassays to ascertain specificity. Incubation of the primary antibody with its immunogenic peptide prevented the labeling of the rat brain (Glavas et al., 2008). In this study, the distribution pattern of the MCH-positive elements observed in the mouse brain was consistent with that described by other groups using other antibodies against MCH (Croizier et al., 2010).

### Double fluorescent immunohistochemistry

The native fluorescence for ChR2-YFP was directly observed in the nodose ganglion and brainstem. In peripheral tissues, YFP was indirectly detected using an antiserum against GFP. After rinsing in phosphate-buffered saline (PBS), free-floating brain sections, whole mounts, or histological slides were incubated with primary antibodies [diluted in 3% normal donkey serum (NDS; Jackson ImmunoResearch, West Grove, PA) and 0.3% Triton X-100 in PBS] at room temperature for 16–24 h. After several PBS washes, the tissues were then incubated with 1:500 to 1:1000 dilutions of the appropriate secondary antibodies for 1 h. The tissue was then covered with Vectashield hard-set mounting medium with DAPI (H1500) and coverslipped. **Table 1** summarizes the combinations of primary and secondary antibodies used in this study. Immunohistochemistry was performed at room temperature with gentle horizontal shaking.

### In situ hybridization

The tissue was processed for chromogenic ISH using the RNAscope 2.5 HD Assay (Brown) from Advanced Cell Diagnostic (Newark, CA). The hypothalami and left nodose ganglia from fed and fasted C57Bl/6 mice were rapidly removed and directly frozen on a bed of dry ice. Fresh-frozen tissue was sectioned at 14 μm using a cryostat and collected on SuperFrost slides. Following the manufacturer’s protocol, the tissue was fixed in 10% formalin and pretreated with a protease-based solution (pretreatment 4) followed by hybridization at 40°C for 2 h in a humidity oven with the double-Z oligo probes for prepro-CART and prepro-MCH listed in **Table 2**. Signal amplification was achieved using 3,3'-diaminobenzidine (DAB), and the tissue was counterstained with hematoxylin. Finally, hybridized slides were dehydrated in graded alcohols, immerged in xylene, and coverslipped with Permaslip mounting medium (Alban Scientific, St. Louis, MO).

**Table 2.** List of reagents used for ISH (RNAscope probes from ACD)

**Gene**	**Accession no.**	**Target region**	**Catalog no., channel**	**Chromogenic label**
CARTPT	NM_013732.7	11–860	432001-c1	DAB, brown
PMCH	NM_029971.2	4–652	478721-c1	DAB, brown

### Confocal imaging and photomicrograph production

Samples from Na_v_1.8-Cre-ChR2-YFP mice were scanned using a Leica SP5 confocal microscope. Oil immersion 40× and 63× objectives were used. The gain and laser intensity were slightly adjusted for each tissue; however, the same scanning parameters were applied when comparing the same tissues from different feeding groups. The line average was 8 or 16. The step increase in the *z*-axis was usually 0.35 µm, and the total number of optical sections in each stack was 10–20. Most of the images in the study were merged into one color and projected onto a single plane, unless mentioned otherwise in the legend. Single optical sections were used for the estimates and colocalization studies. ImageJ (http://rsb.info.nih.gov/ij/) was used to convert all our confocal-acquired images (lif format) into TIFF images (RGB, 300 dpi) and generate scale bars. CART-positive cells were subjectively evaluated by scaling cell profiles clearly containing immunoreactivity within the outline of the YFP-labeled structures that conformed well to the shapes of these structures.

Adobe Photoshop CS5.1 (Adobe Systems, San Jose, CA) was used to combine the digital images into annotated plates. In some instances, several images were stitched together, as indicated in the legend. The contrast and brightness were uniformly adjusted by adding one adjustment layer. Finally, DAPI-labeled structures were converted to grayscale for better contrast, and red–green fluorescence images were converted to magenta–green for colorblind readers.

#### Estimates of double-labeled cells and axons

We estimated the percentage of ChR2-YFP–labeled vagal afferents by counting the cell profiles that were double labeled for peripherin and ChR2-YFP. Likewise, we counted the neurons that were double labeled for CART and ChR2-YFP in the nodose ganglion. A neuron was considered double labeled when the shape of the YFP-positive profile corresponded to that of the CART or peripherin-stained profiles. The neurons were counted in digital images obtained with a Zeiss microscope (Imager ZI, 20× objective) equipped with a scanning stage and attached to the ApoTome system and a digital camera (Axiocam MRm). A desktop computer running Axiovision 4.7 was used to produce the digital images. The neurons in a 1:5 series of sections across the nodose ganglion were counted by a blinded observer. To evaluate the relative intensity of fluorescence in CART-labeled cells, we used a method described in the literature (Fang et al., 2002) with slight modifications. Using ImageJ, the mean absorbance of outlined CART-positive profiles was measured using measurement tools. The average of the mean absorbance of one negative profile in each tissue section of chow-fed mice was used as our 0% intensity (*a*). The average of the mean absorbance of one most intensely stained proﬁle in each tissue section of chow-fed mice was used as 100% intensity (*b*). The intensity of the measured profiles was scaled between the minimal and maximal values as follows:

Relative intensity of CART-labeled profile=(measured mean−a)/(b−a)×100.

The measurements were repeated in a large number of individual cell profiles as indicated in our graph. Data were expressed as the relative intensity (%) of individual CART cells compared to the darkest-stained cells. This method allowed us to easily visualize the full range of immunoreactivity of CART cells across the different feeding groups. Cell profiles were categorized according to their relative immunoreactivity as containing low (0 to <25%), medium (25 to <75%), or high (75 to >100%) immunoreactivity. Finally, we calculated the frequency of neurons falling into each category across feeding groups. None of the estimates described above were meant to provide absolute counts, but rather relative estimates across different feeding conditions. Thus, we did not correct our data for double counting and did not use the stereological technique. The graphs were constructed using GraphPad Prism 7.01.

The estimates described below were performed in a 1:5 series of sections using digital images of single optical sections acquired with a confocal microscope (63×, zoom 6). Counting was performed by a blinded experimenter. The percentage of CART-positive varicosities in the area postrema (AP) was evaluated. Double-labeled varicosities were considered when CART staining was clearly contained within the boundaries of each ChR2-YFP–labeled varicosity. Second, the diameters of the ChR2-YFP–labeled axons and varicosities in the AP were evaluated. The counts and measurements were collected using ImageJ measuring tools.

Immunofluorescence in the rat was captured using a digital camera (Axiocam) attached to a Zeiss microscope Imager ZI equipped with the Apotome system. Several images of the rat nodose ganglion taken at 40× were stitched together using Axiovision 4.7 software. The density of CART-positive cells in the rat nodose ganglion was evaluated using Axiovision 4.7. A blinded observer manually counted the number of cell profiles that were immunoreactive for CART. The number of profiles was divided by the surface of tissue occupied by the nodose ganglion.

ISH signals for prepro-CART mRNA were analyzed on DAB-labeled ganglia counterstained with hematoxylin. Images were taken using the bright-field optics of the Zeiss Axioskop 2 microscope and Axiovision 4.8 software. The percentage of prepro-CART–expressing profiles was estimated by manually counting clearly identifiable neuronal profiles with or without DAB. The intensity of the hybridization signals was further evaluated by subjectively scaling neuronal profiles with low (<20 DAB-positive dots per profile), medium (dense accumulation of DAB with large areas of the cytoplasm still visible), or high (dense accumulation of DAB covering almost entirely the cell profile) signals. We then calculated the frequency of profiles falling into each category across feeding groups.

## Results

### Tracing the vagal afferents using Na_v_1.8-Cre-ChR2-YFP mice

**Figure 1.**
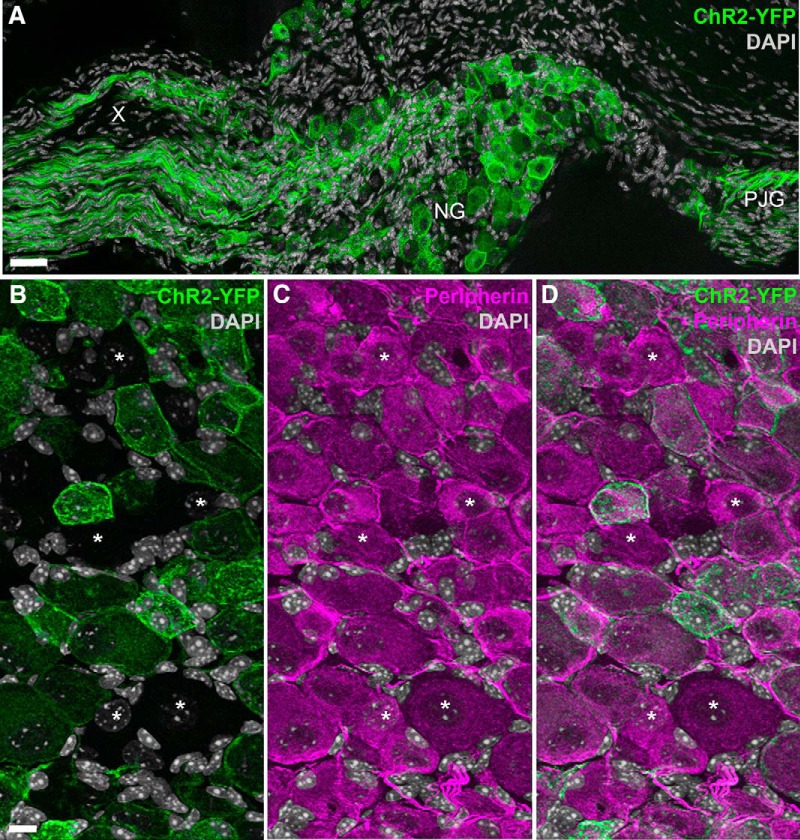
Distribution of ChR2-YFP fluorescence in the nodose ganglion of Na_v_1.8-Cre-ChR2-YFP mice. ***A***, Many neuronal cell bodies and axons were brightly fluorescent in the nodose ganglion (NG) and petrosal-jugular ganglion (PJG) (three images were horizontally stitched together). ***B–D***, Details of NG peripherin-labeled neurons (Alexa Fluor 594). ChR2-YFP is apparent in the membrane and cytoplasm of many NG neurons (but not all) (***B***). Asterisks are positioned over representative peripherin-positive neurons that were not labeled with ChR2-YFP (***B***, ***C***). X, cervical vagus nerve. Scale bars, 40 μm in ***A***; 20 μm in ***B*** (also applies to ***C***, ***D***).

We first tried to establish the usefulness of Na_v_1.8-Cre-ChR2-YFP mice in labeling vagal C-fibers. In the nodose ganglion, the soma of the vagal afferents and their proximal axons showed robust endogenous green fluorescence (**Fig. 1*A***). ChR2-YFP was clearly visible, without the need for immunostaining, in the membrane and cytoplasm of many vagal afferents (**Fig. 1*A*,*B***). We estimated that 82 ± 2% (*n* = 4) of the vagal afferents that stained for peripherin, a panneuronal marker in the peripheral nervous system (Gorham et al., 1990), were also positive for ChR2-YFP (**Fig. 1*B–D***).

**Figure 2.**
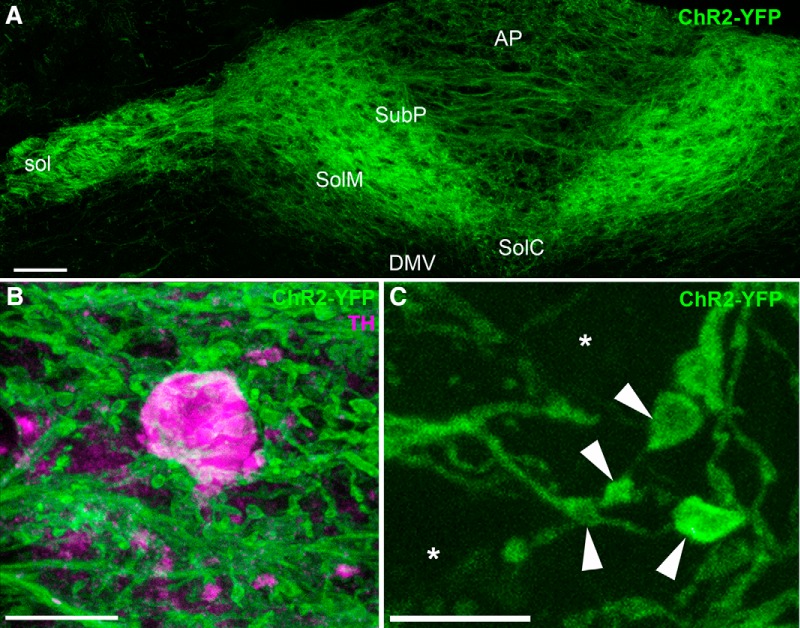
Distribution of ChR2-YFP fluorescence in the dorsovagal complex of Na_v_1.8-Cre-ChR2-YFP mice. ***A***, Vagal afferents terminating in the AP and NTS were labeled with ChR2-YFP (three horizontally stitched images). ***B***, Highly varicose ChR2-YFP–labeled axons circling the cell body of one TH-positive neuron (Alexa Fluor 594) located in the NTS. ***C***, Single optical section of ChR2-YFP–labeled axons in the AP revealing thin varicose axons of varying sizes. Asterisks are positioned over the presumptive locations of the postsynaptic cell bodies, whereas arrowheads indicate representative vagal varicosities. DMV, dorsal nucleus of the solitary tract; sol, solitary tract; commissural part of the nucleus of the solitary tract; SolDM, dorsomedial part of the nucleus of the solitary tract; SolM, intermediate part of the nucleus of the solitary tract; SubP, subpostrema area. Scale bars, 60 μm in ***A***; 5 μm in ***B*** and ***C***.

**Figure 3.**
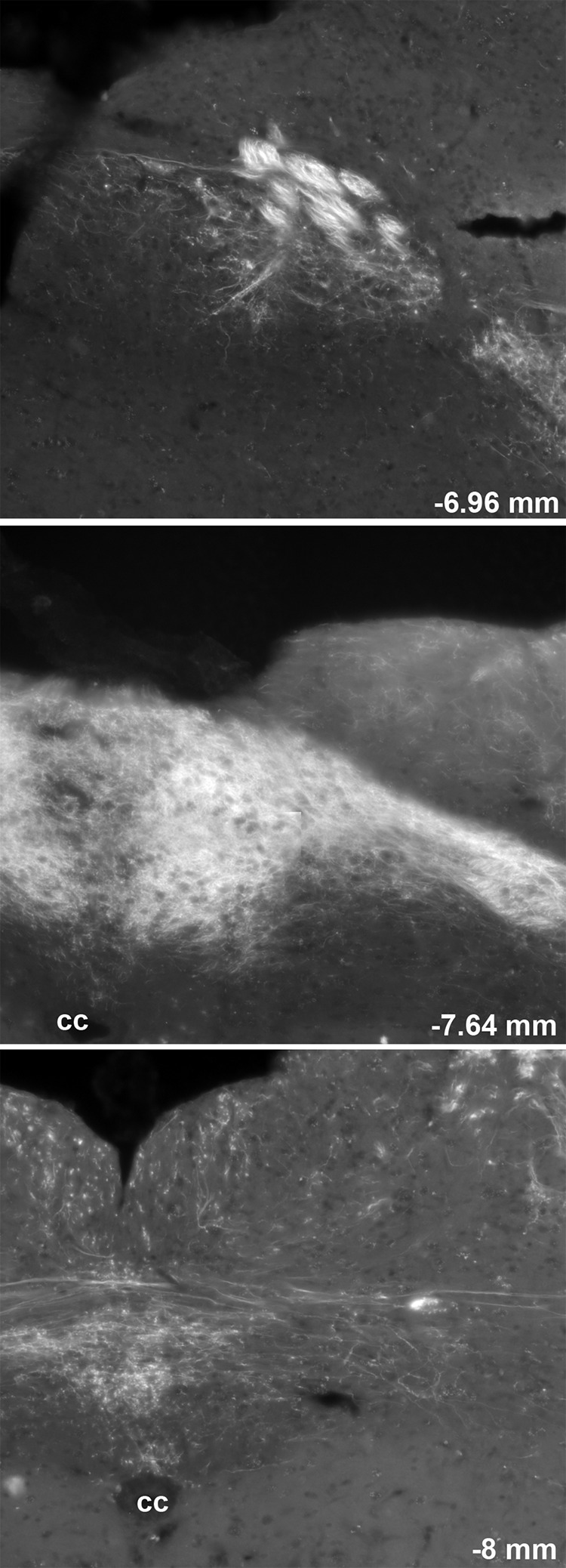
Distribution of ChR2-YFP fluorescence throughout the rostrocaudal extension of the dorsovagal complex of one representative Na_v_1.8-ChR2-YFP mouse. Several digital images were stitched together (epifluorescence with Apotome filter). The number in the bottom right corner indicates the approximate distance from Bregma according to the *Franklin and Paxinos Mouse Brain Atlas* (Third Edition). 4v, fourth ventricle; cc, central canal.

In addition, ChR2-YFP labeled the membranes of the vagal terminals within the dorsovagal complex (**Figs. 2*A*, 3** ). Although we observed a few isolated ChR2-YFP–labeled neurons in the forebrain (mainly in the cortex and striatum), no neurons were detected in the hypothalamus or brainstem (not shown). Thus, the ChR2-YFP–labeled fibers observed in the dorsovagal complex were essentially derived from the nodose ganglion. The densest innervation was observed in the dorsal and medial parts of the nucleus of the solitary tract (NTS), as well as the solitary tract itself (**Fig. 2*A***). Innervation was less dense in the AP and other parts of the NTS (**Fig. 2*A***). The innervation of the dorsovagal complex was very dense, forming bundles of intermingled axons circling the putative location of the postsynaptic neurons. For example, ChR2-YFP varicosities accumulated in the vicinity of TH-positive neurons in the AP and NTS (**Fig. 2*B***). Although it was difficult to distinguish individual axons and varicosities at low magnification, optical sectioning at higher magnification (63×) revealed individual thin axons (average diameter 0.3 ± 0.0 μm; *n* = 3) decorated with boutons and varicosities resembling presynaptic terminals (average diameter 0.9 ± 0.0 μm; *n* = 3) (**Fig. 2*C***). **Movie 1** is a file compilation of a *z*-stack of ChR2-YFP–labeled axons (green) around one TH-positive neuron (red).

Movie 1. File compilation of a *z*-stack of ChR2-YFP–labeled axons (green) around one TH-positive neuron (Alexa Fluor 594) in the AP of the Na_v_1.8-Cre-ChR2-YFP mouse. Note the varicosities in close apposition to the outline of the TH-positive neuron. 10.1523/ENEURO.0174-16.2016.video.1

**Figure 4.**
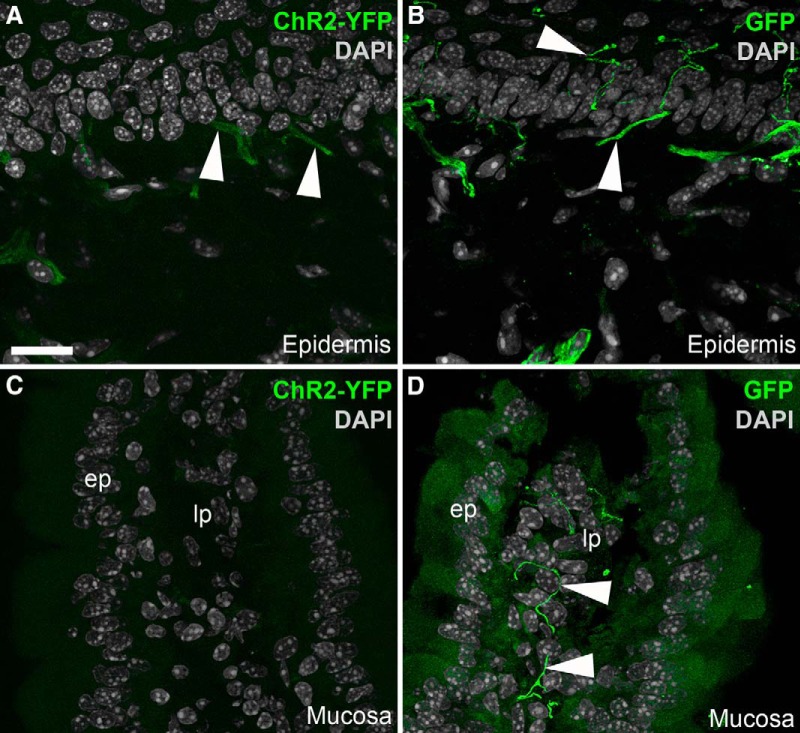
Identification of ChR2-YFP–positive fibers in peripheral tissues from Na_v_1.8-Cre-ChR2-YFP mice. ***A***, Weakly fluorescent structures resembling nerve endings (arrowheads) were observed in the epidermis of the forepaw. ***B***, GFP immunolabeling greatly enhanced the labeling of individual axons ramifying into the epidermis. ***C***, We could not detect endogenous YFP fluorescence in the duodenal mucosa before immunolabeling. ***D***, YFP-labeled axons were evident in the lamina propria (lp) when the tissue was stained for GFP. The antibody produced a small amount of nonspecific background staining in the epithelium (ep). Arrowheads indicate representative YFP-positive fibers. Scale bars, 20 μm in ***A*** (also applies to ***B–D***).

ChR2-YFP was also transported toward peripheral tissues that were innervated by C-fibers (**Fig. 4**). However, the native fluorescence for ChR2-YFP was much weaker in the periphery than in the ganglia or brain. For instance, low to nearly undetectable levels of endogenous YFP fluorescence were seen in the epidermis (**Fig. 4*A***) and intestinal mucosa (**Fig. 4*C***). Immunostaining with an anti-GFP antiserum successfully marked axons and specialized sensory endings in those tissues (**Fig. 4*B*,*D***). Thus, the subsequent experiments depicting ChR2-YFP in the peripheral tissues were performed using GFP immunofluorescence. Overall, Na_v_1.8-Cre-ChR2-YFP mice allowed assessment of the detailed morphology of C-fibers in many visceral tissues (not shown). Importantly, no cell bodies were ever labeled in peripheral organs.

### Distribution and regulation of in vagal afferents of metabolically challenged mice

**Figure 5.**
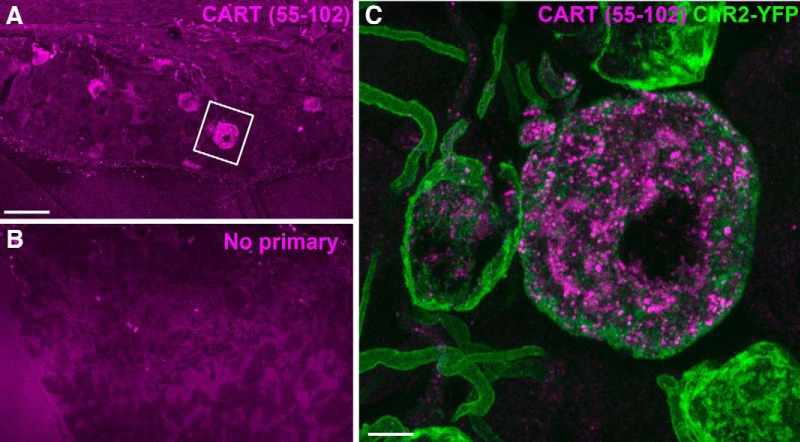
CART(55–102) detection in the nodose ganglion (NG) of Na_v_1.8-Cre-ChR2-YFP mice. ***A***, CART-positive cells (Alexa Fluor 594) resembling neurons were observed throughout the NG. The two cells located in the inset are represented at high magnification in ***C***. ***B***, Omission of the primary antibody eliminated CART immunoreactivity. Only a few debris and background could be observed in the NG. ***C***, Two adjacent ChR2-YFP-, CART-positive neurons are shown. CART is detected in vesicle-like structures in the cytoplasm and proximal axon. The larger neuron contained more immunoreactivity than the smaller neuron. Two other YFP-labeled neurons did not contain CART. Scale bars, 50 μm in ***A*** and ***B***; 5 μm in ***C***.

**Figure 6.**
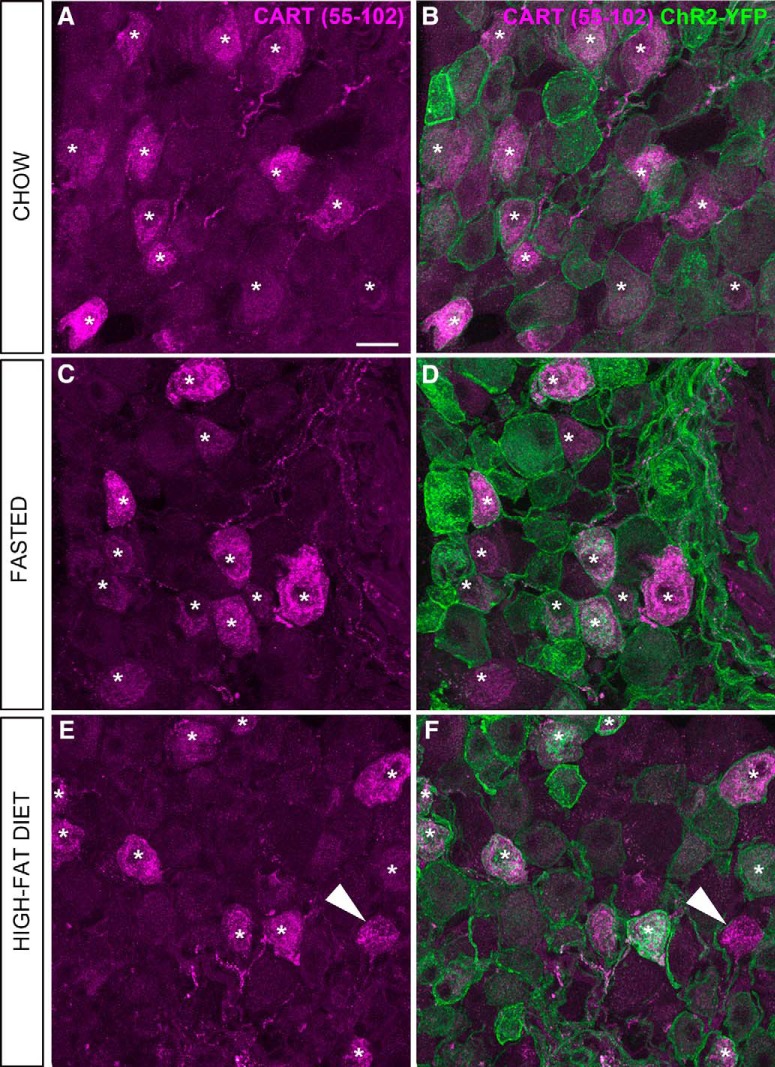
CART(55–102) immunolabeling of the nodose ganglion (NG) of metabolically challenged Na_v_1.8-Cre-ChR2-YFP mice. ***A***, ***C***, ***E***, CART-positive perikarya (Alexa Fluor 594) across the feeding groups. ***B***, ***D***, ***F***, CART staining and endogenous ChR2-YFP fluorescence delineate the outline of the vagal afferents. Asterisks are positioned over representative CART-positive cells. Note that CART immunoreactivity is almost always contained within the cell membrane of the YFP-labeled cells, with a few rare exceptions (arrowhead). By visual inspection, the CART distribution pattern and intensity appeared comparable across the feeding groups. Scale bars, 20 μm in ***A***; applies to all images.

**Figure 7.**
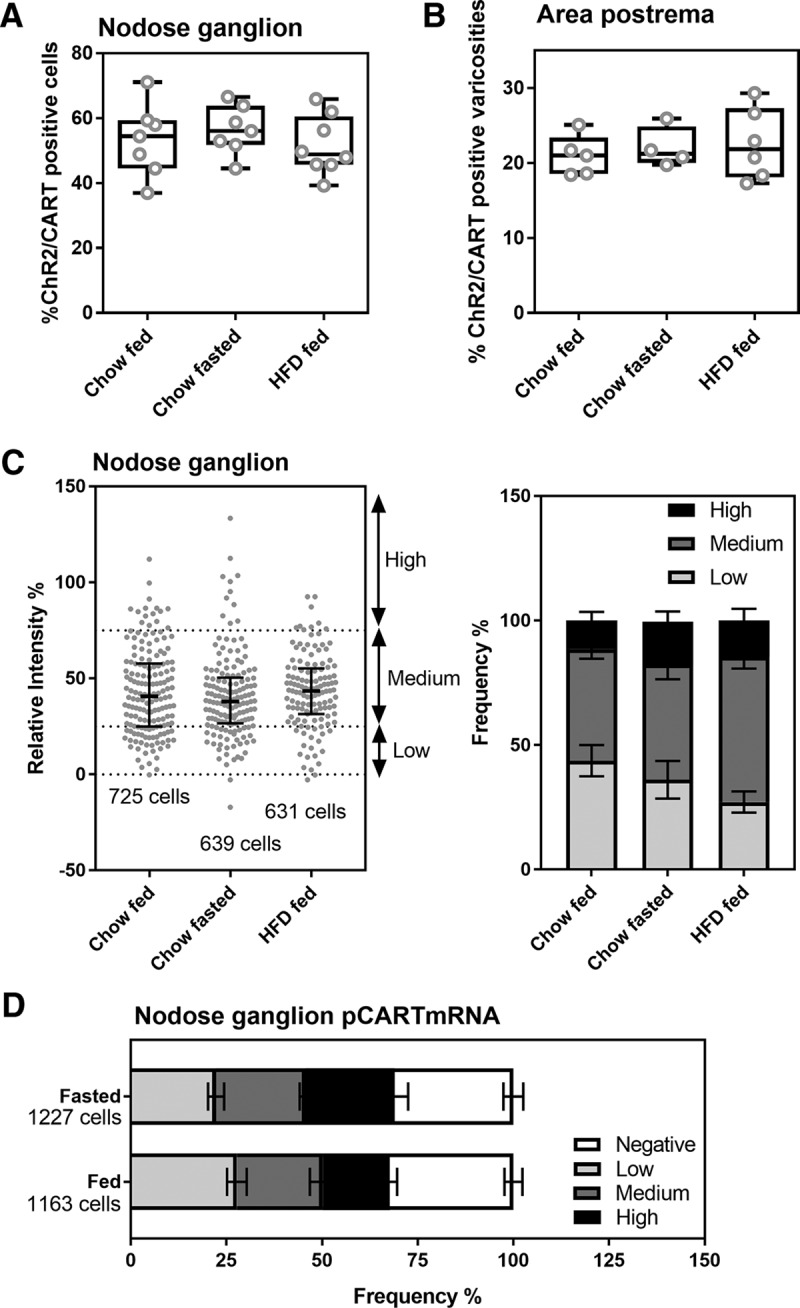
Estimates of CART-positive neurons and afferents across the feeding groups. ***A***, The percentage of YFP- and CART-positive neurons in the nodose ganglion (NG) of Na_v_1.8-Cre-ChR2-YFP mice was not influenced by feeding status (*n* = 7–8 per group). ***B***, The percentage of CART-positive varicosities in the AP was identical in Na_v_1.8-Cre-ChR2-YFP mice under different feeding conditions. Data are expressed as the average of the mean percentage ± maximum values (*n* = 7–8 per group). Each circle represents one value from one mouse. ***C***, Frequency of CART immunoreactivity profiles with varying intensity across feeding groups (*n* = 7 per group). On the left, frequency scattergraph of distributions of individual CART-positive cell profiles with median and interquartile range. Each gray dot is one cell profile. The total number of profiles examined in each group is annotated. On the right, the same data were represented as stacked bars after categorizations of immunoreactivity level. Data are provided as mean percentage ± SEM. ***D***, Frequency of cells expressing pCART mRNA in the nodose ganglia of fed (*n* = 4) and fasted (*n* = 3) wild-type mice. Cells were scaled based on the intensity of hybridization signals. The total number of profiles examined in each group is annotated. Stacked bars provide mean percentage ± SEM.

In the nodose ganglion of Na_v_1.8-Cre-ChR2-YFP mice, immunoreactivity for CART(55–102) was detected in the soma and proximal axons of many neurons (**Figs. 5*A*,*C*, 6**). The nodose ganglion neurons were not stained when the CART primary antibody was omitted (**Fig. 5*B***). In agreement with previous works (Broberger et al., 1999; Zheng et al., 2002; Scruggs et al., 2003), immunoreactivity for CART was evident in large puncta around the cell nucleus and within the proximal axon (**Fig. 5*B***). Approximately 55% of ChR2-YFP–labeled neurons were CART-positive (**Figs. 6**, **7*A***), and >99% of CART-positive cells were labeled with ChR2-YFP. CART staining in the nodose ganglion looked identical in fed, fasted, and obese animals when observed under blinded conditions (**Fig. 6*A–F***). According to our estimates, the percentage of ChR2-YFP–labeled neurons containing CART was identical across the feeding groups (**Fig. 7*A***). Because CART immunoreactivity varied in intensity between neurons, we also examined the relative intensity of CART(55–102) immunoreactivity in a large number of cell profiles (**Fig. 7*C***). The percentages of cells with low, medium, and high immunoreactivity were identical across the feeding groups (**Fig. 7*C***).

**Figure 8.**
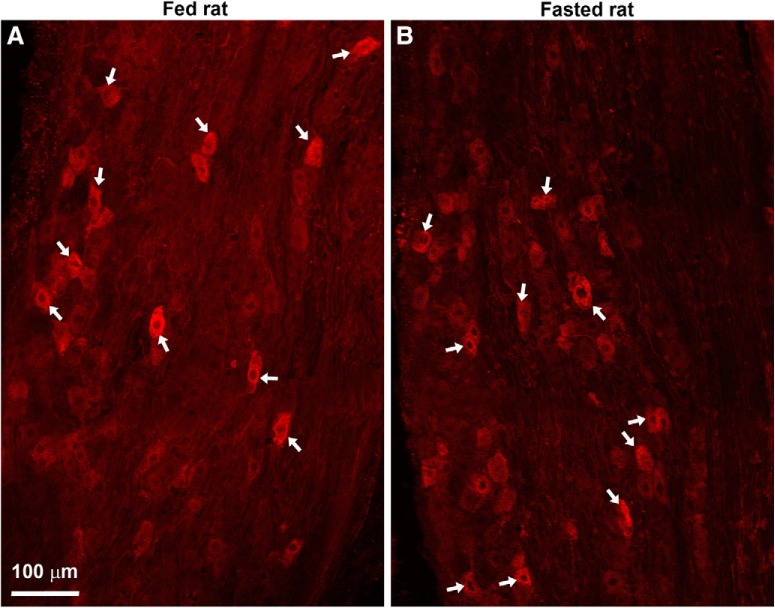
***A***, ***B***, CART(55–102)-positive neurons in the nodose ganglia of lean Zucker rats. Several digital images were stitched together (epifluorescence with Apotome filter). Many CART-positive neurons (Alexa Fluor 594) were observed in the nodose ganglion of fed and fasted rats. White arrows indicate examples of CART neurons. Scale bar, 100 μm in ***A***; applies to ***B***.

Most of the studies on the impact of fasting on vagal CART have been performed in rats. To rule out the possibility of species differences, we examined CART in fed and fasted rats (**Fig. 8**). The distribution and intensity of CART staining in the nodose ganglion looked identical in fed and fasted rats when observed under blinded conditions (**Fig. 8**). Moreover, the density of CART-positive profiles across the ganglion was not significantly different in fed (125 ± 18 cells/mm^2^, *n* = 3) and fasted (160 ± 27 cells/mm^2^, *n* = 3) rats.

**Figure 9.**
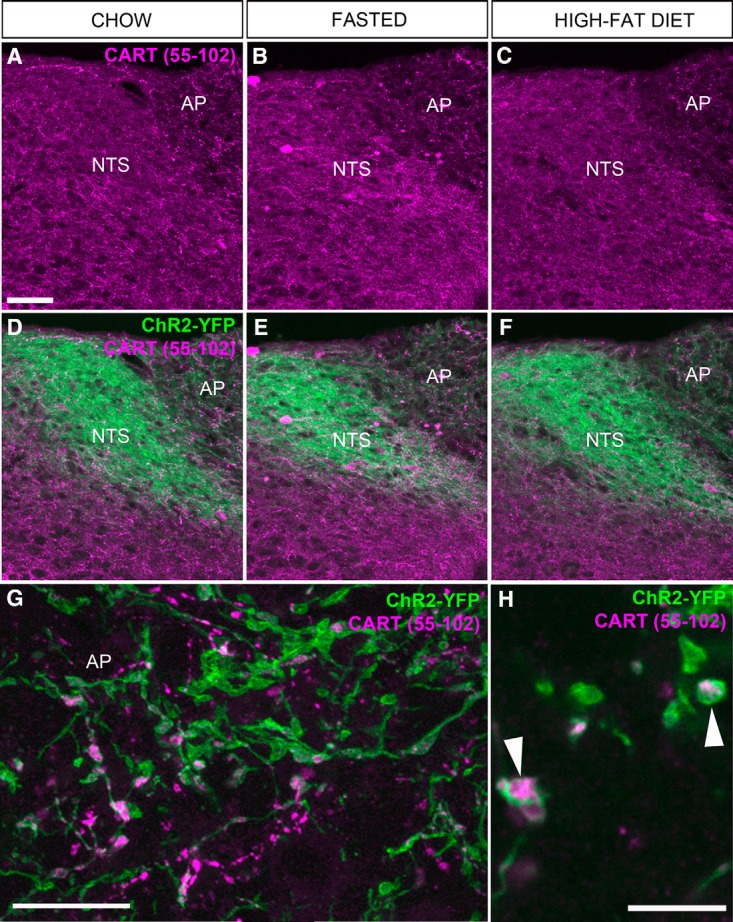
CART(55–102) immunolabeling of the dorsovagal complex of metabolically challenged Na_v_1.8-Cre-ChR2-YFP mice. ***A***, ***B***, ***C***, CART-positive (Alexa Fluor 594) perikarya and fibers of various origins were observed throughout the NTS and AP. We noticed a trend toward increased immunoreactivity in the fasted groups. However, our estimates (see Fig. 6) indicate that the levels of CART in vagal afferents remained unchanged across feeding conditions. ***D***, ***E***, ***F***, CART immunoreactivity combined with endogenous ChR2-YFP fluorescence. ***G***, CART was very abundant in the AP. In particular, many YFP-labeled varicosities of vagal origin were enriched for CART. However, we also observed CART immunoreactivity that was not contained in ChR2-YFP–labeled fibers. ***H***, High magnification of the distribution of CART immunoreactivity in the vagal fibers of the AP in a single optical plan. Of note, CART frequently labeled the cytoplasm contained within the YFP-labeled varicosities, which are indicated by arrowheads. Scale bars, 60 μm in ***A***, applies to ***B–F***; 15 μm in ***G***; 5 μm in ***H***.

We also assessed CART immunoreactivity in the vagal terminals in the dorsovagal complex. CART(55–102) immunoreactivity was observed throughout the entire dorsovagal complex and included both neuronal soma and fibers (**Fig. 9*A***). Across the feeding groups, the distribution and intensity of CART immunoreactivity was directly comparable (**Fig. 9*A–C***). CART immunoreactivity was observed in ChR2-YFP axons and varicosities in the AP and commissural part of the nucleus of the solitary tract (SolC), in agreement with the results of Zheng et al. (2002). In these regions, CART immunoreactivity corresponded well to the shape of the YFP-labeled varicosities (**Fig. 9*G*,*H***). However, not all CART-positive elements corresponded to YFP-labeled structures, and in other parts of the dorsovagal complex, double-labeled axons were rarely observed (not shown). In the AP, CART(55–102) was present in approximately 22% of the ChR2-YFP–labeled varicosities (**Fig. 7*B***). In agreement with our results in the nodose ganglion, our estimates showed identical percentages of varicosities that were colabeled for CART and ChR2-YFP in the AP of the fed, fasted, and high-fat-diet–fed animals (**Fig. 7*B***). We did not notice obvious differences in the shape, amount, and distribution of ChR2-YFP–labeled fibers across the feeding groups (**Fig. 9*D–F***).

**Figure 10.**
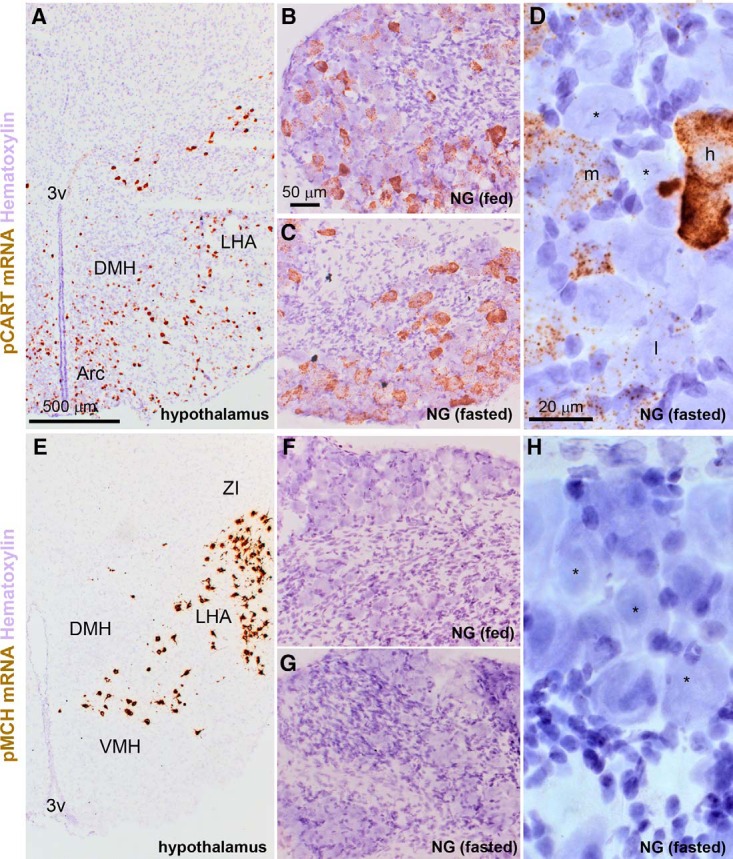
Detection of prepro-CART (pCART) and prepro-MCH (pMCH) mRNAs using chromogenic ISH. ***A***, pCART hybridization signals (brown DAB; bright-field optics) were strong in select hypothalamic nuclei. ***B***, Throughout the nodose ganglion of fed mice, pCART signals of varying intensity were observed in many cell profiles. ***C***, The nodose ganglion of fasted mice also contained pCART hybridization signals. ***D***, Details of the hybridization signal in the nodose ganglion of one fasted mouse. Please note representative cell profiles without signal (*), or with low (l), medium (m), and high (h) signals. ***E***, Hybridization signals for pMCH were very strong in neurons of the lateral hypothalamus. ***F***, ***G***, In contrast to the hypothalamus, pMCH signals were not observed in the nodose ganglia of fed and fasted mice. ***H***, Details of the nodose ganglion of one fasted mouse showing several neuronal profiles completely devoid of signals (*). Tissue was counterstained with hematoxylin. 3V, third ventricle; Arc, arcuate nucleus; DMH, dorsomedial hypothalamus; LHA, lateral hypothalamus; NG, nodose ganglion; VMH, ventromedial hypothalamus; ZI, zona incerta. Scale bars, 500 μm in ***A*** and ***E***; 50 μm in ***B***, ***C***, ***F***, and ***G***; 20 μm in ***D*** and ***H***.

Finally, we assessed the expression of prepro-CART mRNA in the nodose ganglia of fed and fasted wild-type mice. Using chromogenic ISH, prepro-CART mRNA was detected at high levels in select hypothalamic nuclei known to express CART (**Fig. 10*A***). Robust signals for prepro-CART mRNA were also present in nodose ganglia of fed and fasted mice (**Fig. 10*B*,*C***). Variable amounts of DAB accumulated in the soma of many vagal sensory neurons, including in the fasted condition (**Fig. 10*D***). The percentage of prepro-CART–expressing cells and the relative intensity of hybridization signal per cell did not significantly change after fasting (**Fig. 7*D***).

### Absence of MCH in vagal afferents of metabolically challenged mice

**Figure 11.**
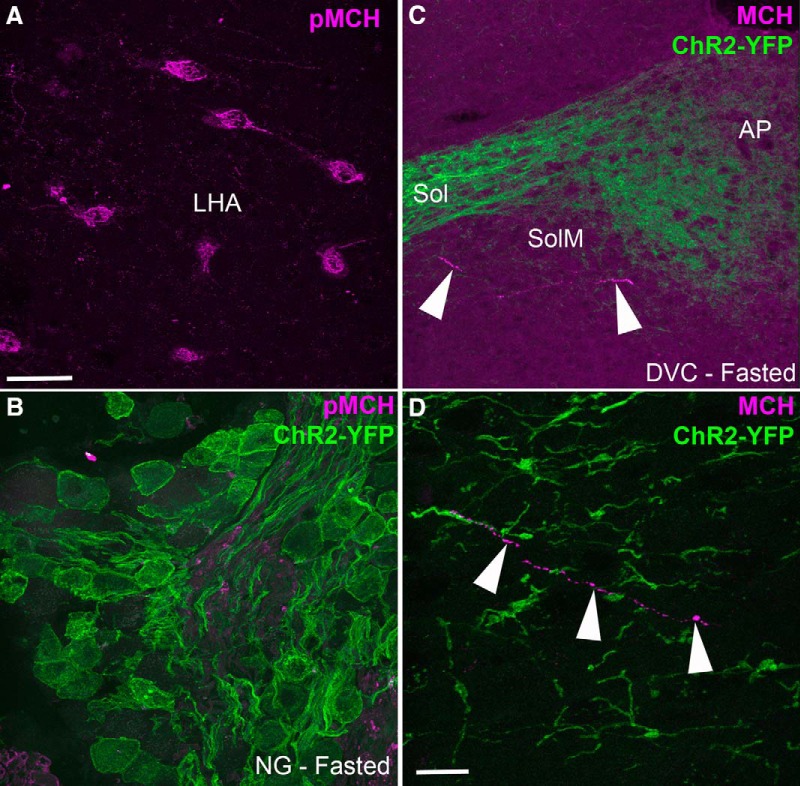
Absence of MCH staining in the vagal afferents of metabolically challenged Na_v_1.8-Cre-ChR2-YFP mice. ***A***, An antiserum against prepro-MCH (pMCH) labeled perikarya (Alexa Fluor 594) in the lateral hypothalamus (LHA). ***B***, PMCH was undetectable in the nodose ganglion (NG) of fasted Na_v_1.8-Cre-ChR2-YFP mice. ***C***, ***D***, An antiserum against the MCH peptide labeled very few axons (arrowhead) in the medial NTS (SolM). MCH immunoreactivity was never observed in the YFP-labeled fibers of vagal origin. Arrowheads point to one MCH-positive axon. sol, solitary tract. Scale bars, 40 μm in ***A*** and ***B***; 10 μm in ***C***.

Because MCH has been reported to be produced in CART afferents in response to fasting, we sought to evaluate the presence of both pro-MCH and MCH in our mice. First, we used an antiserum against pro-MCH that preferentially stained the perikarya. We successfully detected immunoreactivity for prepro-MCH in the perikarya of neurons in the lateral hypothalamus (**Fig. 11*A***). The lateral hypothalamus neurons were not stained when the pro-MCH antibody was omitted (not shown). We were unable to see any immunoreactivity for pro-MCH in the nodose ganglion of Na_v_1.8-Cre-ChR2-YFP mice, even in the fasted mice (**Fig. 11*B***). Identical results were obtained in samples incubated with higher concentrations of antibody (up to 1:100; data not shown). Second, we used an antiserum against MCH itself. MCH immunoreactivity was observed in axons across the forebrain; however, the dorsovagal complex of the fed, fasted, and obese animals contained very little MCH (**Fig. 11*C*,*D***). Typically, isolated MCH-positive varicose axons were observed in the ventral region of the medial part of the nucleus of the solitary tract (SolM), where the YFP-labeled fibers were relatively sparse (**Fig. 11*C*,*D***). These MCH-positive axons never colocalized with YFP-labeled axons, further confirming that vagal afferents do not produce MCH. To rule out the possibility of species differences, we also examined pro-MCH in the nodose ganglion of fed and fasted rats. Consistent with our mouse data, we were not able to detect immunoreactivity (data not shown).

To rule out the possibility of a false-negative result, we also assessed the expression of prepro-MCH mRNA in the nodose ganglia of fed and fasted wild-type mice. As expected, chromogenic ISH revealed very intense signals in the lateral hypothalamus (**Fig. 10*E***). In contrast, the rest of the brain and the nodose ganglion were completely devoid of signal, regardless of the nutritional status of the mice (**Fig. 10*F–H***).

### Absence of CART immunoreactivity in gastrointestinal endings

**Figure 12.**
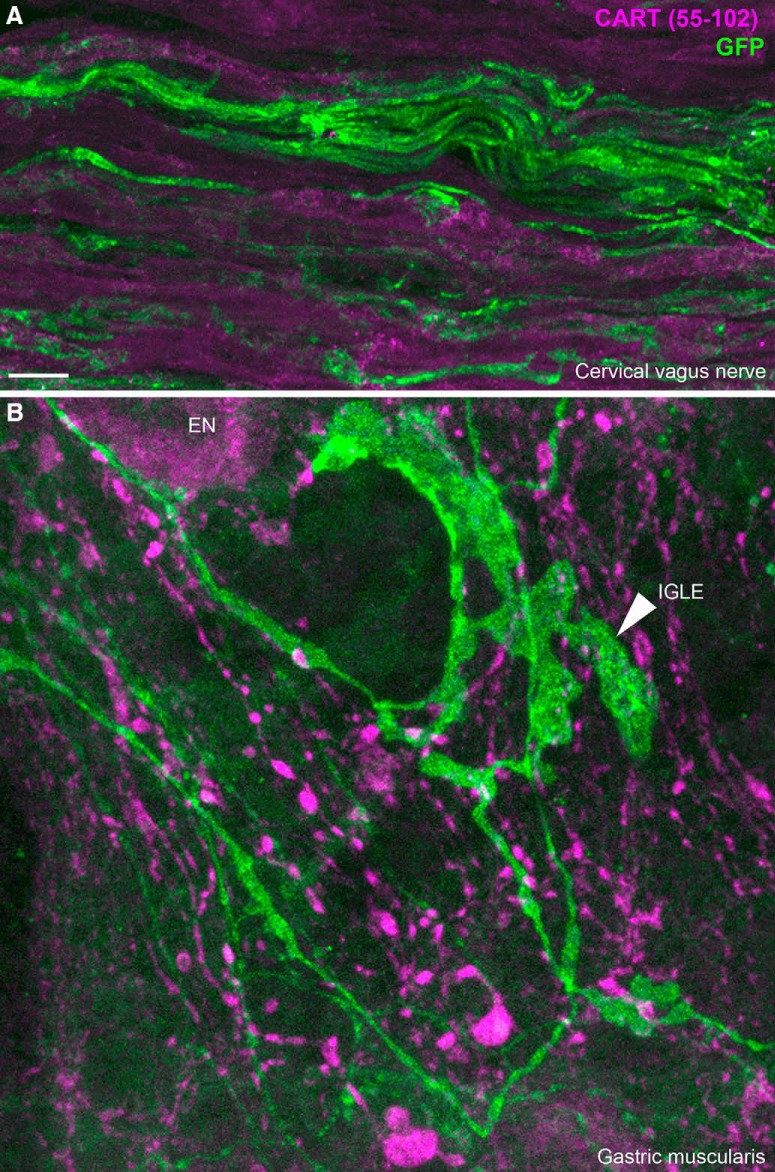
CART(55–102) immunolabeling of peripheral ChR2-YFP–labeled fibers. ***A***, ChR2-YFP fibers contained within the cervical vagus nerve. CART was not detected in the vagus nerve. We obtained similar results in the cervical and subdiaphragmatic nerves on both sides. ***B***, In whole mounts of the gastric muscularis, numerous GFP-positive axons and specialized endings can be observed. The leafy structure may correspond to an intraganglionic laminar ending (arrowhead) of vagal origin. CART immunoreactivity was abundant in large varicose fibers and, to a lesser extent, the cell body of enteric neurons (EN). Nonetheless, CART immunoreactivity was not found in GFP-stained fibers. IGLE, intraganglionic laminar ending; EN, enteric neuron. Scale bar, 10 μm in ***A*** and ***B***.

In the vagus nerve itself, we did not observe CART immunoreactivity in any YFP-labeled axons (**Fig. 12*A***). In the gastrointestinal tract, abundant CART immunoreactivity was observed, likely originating from postganglionic neurons (Calupca et al., 2001; Ekblad et al., 2003; Gonsalvez et al., 2010) and vagal efferents (Zheng et al., 2002). In those tissues, CART staining was observed in enteric neurons and fibers running closely to ChR2-YFP axons. However, CART did not colocalize with YFP-labeled axons themselves. For instance, ChR2-YFP–labeled specialized endings resembling vagal intraganglionic laminar endings were CART negative (**Fig. 12*B***). Similar observations were reported in the duodenal mucosa (not shown). This suggested that CART is not efficiently transported to the peripheral sensory endings of C-fibers, including those in the vagus nerve.

## Discussion

The present study characterized CART(55–102) immunoreactivity in the vagal afferents of Na_v_1.8-Cre-ChR2-YFP mice. Our results indicated that CART(55–102) is constitutively expressed in approximately 55% of vagal Na_v_1.8-expressing neurons, even in fasted mice. MCH, a peptide that was previously reported to be induced in CART-positive vagal afferents of fasted animals, was undetectable. In contrast to several studies, we concluded that the neuropeptidergic profile of the CART-positive vagal afferents is remarkably stable across the energy balance spectrum.

### Technical considerations

We used reporter mice that invariably express ChR2-YFP in Na_v_1.8-Cre–expressing cells to label C-fibers in their entirety. Na_v_1.8-Cre mice crossed with either tdTomato or ChR2 reporter animals have previously been used to label the perikarya and axons of C-fibers, including somatosensory nociceptors (Gautron et al., 2011; Shields et al., 2012; Daou et al., 2013; Bonin et al., 2016). To our knowledge, this is the first study to use Na_v_1.8-Cre-ChR2-YFP mice to describe visceral C-fibers. Overall, our data further validate the usefulness of ChR2-YFP as an excellent neuronal tracer. In particular, ChR2-YFP presented the advantage of brightly labeling the membrane of both central and peripheral endings, thereby allowing the tracing of specialized endings at high resolution. In the dorsovagal complex, this approach was critical for distinguishing the axons of vagal origins from central axons. A minor caveat of this mouse model is that GFP immunohistochemistry was required to detect ChR2-YFP in peripheral tissues, probably because of its limited peripheral transport. In the past, isolectin B4 binding to C-fiber neurons has been commonly used to trace the perikarya and central axons of C-fiber neurons (Fang et al., 2006; Fullmer et al., 2007; Solorzano et al., 2015). However, because of extraneuronal binding (Kirkeby and Moe, 2001), isolectin B4 does not permit the labeling of axons that innervate peripheral tissues. In addition, numerous developmental, biological, and inflammatory factors influence isolectin B4 binding to sensory neurons (Beland and Fitzgerald, 2001; Fullmer et al., 2007); Therefore, it is possible that isolectin B4 binding to C-fibers may vary in response to different physiological conditions.

In contrast to several previous studies (de Lartigue et al., 2007, 2010,2014), we did not observe any suppression of CART immunoreactivity in response to fasting, even though we used the same antibodies. However, our mouse data are in agreement with our ISH and the results of Broberger et al. (1999), who investigated the regulation of CART mRNA in metabolically challenged rats. Importantly, the number and appearance of CART-labeled cells were consistent with the published literature (Broberger et al., 1999; Zheng et al., 2002; Scruggs et al., 2003). In addition, abundant CART immunoreactivity was observed in the nodose ganglion of fasted rats. Together, these observations made us confident that the CART immunostaining observed in our study, including in the fasted mice, was not the result of false-positive labeling. Likewise, our failure to detect MCH in the nodose ganglion was not likely due to false-negative results because we used well-characterized antisera that successfully stained the brain. If MCH had been truly produced in the nodose ganglion of fasted animals, then we should have been able to detect it in the vagal terminals of the dorsovagal complex. More importantly, prepro-MCH mRNA was completely undetectable in the mouse nodose ganglion, even in the fasted condition. The absence of MCH in vagal afferents is consistent with the fact that the administration of MCH in the fourth ventricle does not influence food intake (Zheng et al., 2005; Baird et al., 2008). Even though differences in age, strain, bleeds of antibody, and diets may have influenced our results, we are confident that MCH is not produced by the vagal afferents.

### Neuropeptidergic makeup of the vagal afferents

Although many different neuropeptides have been described in vagal afferents, the majority of them are expressed at relatively low levels or in small numbers of neurons, which is true for substance P, galanin, neuropeptide Y, and calcitonin gene–related peptide (CGRP; Helke and Niederer, 1990; Calingasan and Ritter, 1992; Kummer et al., 1993; Matsumoto et al., 2003; Young et al., 2008). Of note, the aforementioned peptides are often produced by vagal afferents innervating the lungs (Undem et al., 2004; Plato et al., 2006) and therefore are not necessarily involved in feeding regulation. This contrasts sharply with CART, which is abundantly expressed in many vagal afferents, including those involved in feeding. Furthermore, our results indicate that CART expression in the nodose ganglion is not influenced by metabolic challenges. This result is not entirely surprising, because few physiological or pathophysiological stimuli have been reported to modify neuropeptide expression in vagal afferents, with the notable exception of the peripheral axotomy (Zhang et al., 1996; Reimer and Kanje, 1999). Hence, the neuropeptidergic profiles of the CART-positive hypothalamic neurons and CART-positive vagal afferents are substantially different. For instance, hypothalamic CART neurons often coexpress several feeding-related peptides (Elias et al., 1998). Moreover, hypothalamic CART expression is significantly altered by metabolic challenges, including food restriction (Kristensen et al., 1998) and high-fat-diet feeding (Lee et al., 2013). Nonetheless, even in the hypothalamus, we are not aware of neurons switching from being anorectic to being orexigenic.

Most published studies agree that approximately 40–50% of neurons are immunoreactive for CART in rats and mice in the fed condition (Zheng et al., 2002; de Lartigue et al., 2007; Gautron et al., 2012). Using radioactive ISH, approximately 48% of vagal afferents were found to express CART in the rat (Broberger et al., 1999). However, using chromogenic ISH, we found that approximately 67% of the mouse vagal afferents expressed CART. This is likely because the RNAscope technology is not only more sensitive than immunofluorescence but also provides a better signal-to-noise ratio than radioactive ISH (Wang et al., 2012). Nonetheless, species differences in the distribution and percentage of vagal afferents expressing neuropeptides have been reported in the nodose ganglion of rats and mice (Zhuo et al., 1997). Therefore, we cannot completely rule out that CART mRNA is expressed in more vagal afferents in the mouse than in the rat. However, our current data in both species combined with that of Broberger et al. (1999) support the view that vagal CART is not regulated by fasting.

### Localization of CART in vagal terminals

Many neuropeptides, including vasoactive intestinal peptide and CGRP, are known to be transported from the cell body of vagal afferents to their peripheral terminals (Zhuo et al., 1995). We expected to find CART in C-fiber terminals located in the stomach and duodenum, because previous studies clearly showed that CART-positive vagal afferents project to these locations (Zheng et al., 2002). Surprisingly, we failed to detect CART immunoreactivity in the axons and peripheral endings of vagal neurons that innervate the stomach wall and duodenal mucosa. We concluded that CART was not efficiently transported to the vagal endings and, consequently, may serve mainly as a central neurotransmitter. However, we cannot entirely rule out that our study missed CART immunoreactivity in the gastrointestinal tract. Further tracing studies are therefore warranted to determine the projection sites of CART-positive vagal afferents in both thoracic and abdominal viscera.

## Synthesis

Reviewing Editor: Quentin Pittman, University of Calgary<br />Decisions are customarily a result of the Reviewing Editor and the peer reviewers coming together and discussing their recommendations until a consensus is reached. When revisions are invited, a fact-based synthesis statement explaining their decision and outlining what is needed to prepare a revision will be listed below. The following reviewer(s) agreed to reveal their identity: Keith Sharkey, Thomas Finger. For some manuscripts transferred from JNeurosci, a decision is made to accept the manuscript without peer review and a brief statement to this effect will follow

Both of the reviewers find your manuscript substantially improved as a result of your additional experiments. From my perspective, I believe that your manuscript has moved from one which most likely would have only added confusion to the field to one which might now be the definitive study.

The reviewers and I however feel that a few small tweaks would improve your manuscript even more with attention to the following details and I urge you to incorporate them. They are as follows:

1. The title still is not optimal. The news value of this report is not the relative abundance of the transcript but in their unchanged level in the face of metabolic changes. So something like the following might place the emphasis better:

Levels of Cocaine and Amphetamine-Regulated Transcript in Vagal afferents in the mouse are unaltered in response to metabolic challenges.

2.The odd phrase "across the energy balance spectrum." still appears in the significance statement without explanation.

3.Table 1 would be easier to read and interpret if the rows and columns were switched so that immunogen sequence could appear in a more linear fashion.

4.The images in Fig 3 are not very sharp. The lower left panel, from level -7.2 is especially poor and should be replaced.

Please incorporate these changes and return the manuscript. I will deal with it directly and it will not go out for further review.
